# Integrated Annotation and Analysis of *In Situ* Hybridization Images Using the ImAnno System: Application to the Ear and Sensory Organs of the Fetal Mouse

**DOI:** 10.1371/journal.pone.0118024

**Published:** 2015-02-23

**Authors:** Raymond Romand, Raymond Ripp, Laetitia Poidevin, Marcel Boeglin, Lars Geffers, Pascal Dollé, Olivier Poch

**Affiliations:** 1 Developmental Biology and Stem Cells Department, Institut de Génétique et de Biologie Moléculaire et Cellulaire (CNRS, INSERM, Université de Strasbourg), BP163, 67404 Illkirch Cedex, France; 2 LBGI Bioinformatique et Génomique Intégratives, ICube Laboratory and Strasbourg Federation of Translational Medecine (FMTS), University of Strasbourg and CNRS, Strasbourg, France; 3 Imaging & Microscopy Platform, Institut de Génétique et de Biologie Moléculaire et Cellulaire (CNRS, INSERM, Université de Strasbourg), BP163, 67404 Illkirch Cedex, France; 4 Department of Genes and Behavior, Max Planck Institute for Biophysical Chemistry, Am Faßberg 11, 37077 Göttingen, Germany; University of Washington, Institute for Stem Cells and Regenerative Medicine, UNITED STATES

## Abstract

An *in situ* hybridization (ISH) study was performed on 2000 murine genes representing around 10% of the protein-coding genes present in the mouse genome using data generated by the EURExpress consortium. This study was carried out in 25 tissues of late gestation embryos (E14.5), with a special emphasis on the developing ear and on five distinct developing sensory organs, including the cochlea, the vestibular receptors, the sensory retina, the olfactory organ, and the vibrissae follicles. The results obtained from an analysis of more than 11,000 micrographs have been integrated in a newly developed knowledgebase, called ImAnno. In addition to managing the multilevel micrograph annotations performed by human experts, ImAnno provides public access to various integrated databases and tools. Thus, it facilitates the analysis of complex ISH gene expression patterns, as well as functional annotation and interaction of gene sets. It also provides direct links to human pathways and diseases. Hierarchical clustering of expression patterns in the 25 tissues revealed three main branches corresponding to tissues with common functions and/or embryonic origins. To illustrate the integrative power of ImAnno, we explored the expression, function and disease traits of the sensory epithelia of the five presumptive sensory organs. The study identified 623 genes (out of 2000) concomitantly expressed in the five embryonic epithelia, among which many (∼12%) were involved in human disorders. Finally, various multilevel interaction networks were characterized, highlighting differential functional enrichments of directly or indirectly interacting genes. These analyses exemplify an under-represention of "sensory" functions in the sensory gene set suggests that E14.5 is a pivotal stage between the developmental stage and the functional phase that will be fully reached only after birth.

## Introduction

One of the main challenges of developmental biology is to decipher the interplay between specific pathways and gene networks involved in the different phases of the ontogenesis of an organism. Such an ambitious goal can now be envisioned thanks to the recent biotechnological and bioinformatics developments that are providing massive amounts of gene expression data, as well as the powerful data mining tools available in several atlases dedicated to gene expression in whole organisms or in specific organs/tissues [1,2,3,4,5,6,7,8]. A recent review of web-based gene expression atlases for the mouse stressed the practical usefulness of these approaches [7]. Among these, the GenePaint [9] and EURExpress consortia (http://www.genepaint.org; http://www.eurexpress.org) aim to develop a powerful mouse transcriptome digital atlas, by means of *in situ* hybridization (ISH) in the whole animal at a specific stage of development: embryonic day 14.5. In this context, as members of the consortia, we were responsible for producing a subset of *in situ* hybridization slides, as well as the corresponding micro-photographs, *via* an automated microscopy image acquisition system. Expert anatomists from EURExpress manually annotated the expression profiles for over 18000 protein-coding genes and 1420 anatomic terms. This atlas now provides a semi-quantitative estimate of mRNA abundance, allowing the localization of transcript distributions at the cellular level within anatomical substructures [4,5]. In complement to the EURExpress projects, we have annotated various tissues, corresponding to the eye, teeth, ear and other sensory organs, with additional specific anatomic details. Finally, since the ear has not been studied in great detail in the available expression atlases due to its anatomical complexity, we decided to produce images for 2000 randomly chosen genes representing ∼10% of all murine protein-coding genes [10,11]. Using the existing ISH plates, this led to 11,000 additional ISH micrographs with higher magnification focused on detailed analysis of 25 ear and sensory system tissues or structures of late gestation embryos (E14.5).

To address the challenges involved in performing such a detailed integrated annotation process, we developed the ImAnno knowledgebase (imanno.lbgi.fr) with user-oriented and customizable services to annotate, manage, query and analyze the genes with their ISH expression images. ImAnno is designed as a gene centric database to allow the expert biologist to annotate, with standardized terms, gene expression patterns of detailed tissues using ISH experimental data. ImAnno also provides multi-filter search tools, allowing the user to perform complex combinatorial queries producing lists of genes, which can then be used as input for subsequent integrated analyses linked to external databases related to interactomics, gene ontology, transcriptomic expression, mutation or pathological information. ImAnno was used to annotate eye and teeth images as well as the 2000 randomly chosen genes in the 25 tissues.

In this manuscript, we focus on the 2000 genes to investigate the relationships between the 25 annotated tissues and to explore their common/different functions and/or embryonic origins. A detailed analysis of the sensory region was performed, including the five presumptive sensory organs (KUROV), i.e. the Kölliker’s organ (K) for the cochlea, the utricle sensory region (U) for the vestibular organs, the neural retina (R) for the eye, the sensory region of the olfactory organ (O) and the vibrissae follicles (V) for skin mechano-receptors. This analysis allowed us to identify pathways common to the five sensory organs, and to distinguish pathways related to organ ontogenesis from those that are probably involved in the sensory function at E14.5 and later stages of development. Finally, we illustrate the relationships between gene expression patterns in sensory organs and known human pathologies.

## Materials and Methods

### Tissue description

**Fig 1:**
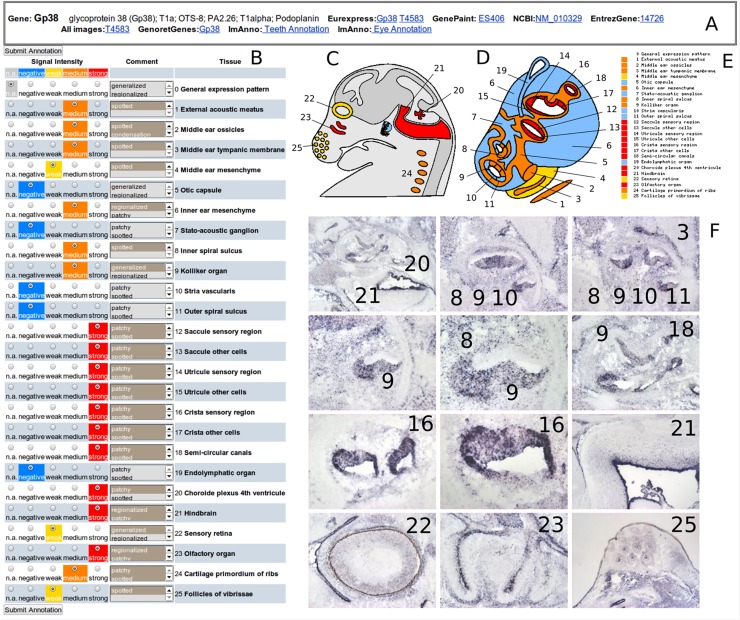
ImAnno web page of the Gp38 gene. A: Upper header provides information about the gene and links to other databases. B: Annotation form with radio buttons to select expression values (n.a.: not analyzed), negative, weak, medium, strong are associated with a blue, yellow, orange, red color code respectively. Predefined keywords or free observations can be added in the Comment boxes. C-E: User accessible web form summarizing the expression features (with the same color codes) in a tissue schema (C, D) with the numbered tissue list (E). F: Overview of the ISH images used to annotate the Gp38 expression pattern with the respective tissue labeling.

Fig. 1 illustrates the tissues or structures annotated by experts, including 6 non-ear tissues: the choroid plexus from the roof of the 4^th^ ventricle as a secretory organ, the hindbrain, retina, olfactory organ, vibrissae follicles and the thoracic rib primordia as non-otic capsule cartilage primordia (Fig. 1C) and 19 ear tissues or structures (Fig. 1D). The external ear is restricted to the external acoustic meatus, while the middle ear includes 3 tissues: ossicles, tympanic membrane and mesenchyme. The inner ear is encapsulated inside the otic capsule and is composed of 2 sensory organs, cochlea and vestibule, and 14 tissues. In the developing inner ear, the ventral region of the basal cochlear canal comprises the inner spiral sulcus and the Kölliker’s organ, which includes the prospective sensory region of the cochlea. The dorsal region of the cochlear canal includes the stria vascularis and the outer spiral sulcus. The vestibule consists of 5 vestibular organs: 3 cristae from the semi-circular canals and 2 maculae from the saccule and the utricle respectively. The stato-acoustic ganglion and the corresponding nerve consist of two components: the auditory nerve innervating the cochlea and the vestibular nerve innervating the five vestibular organs. The endolymphatic system included in the inner ear consists of two components: the duct and the endolymphatic sac.

### Microphotographs from ISH data

Template design was performed by the European GenePaint and EURExpress consortia (http://www.genepaint.org, http://www.eurexpress.org) and template sequences are available at http://www.genepaint.org [9]. No animals were used thereafter for the analytical and bioinformatics work reported in the present manuscript. Methods for animal care, tissue preparation and sectioning of E14.5 mouse fetuses (C57BL/6J strain) are described in detail in [5, 12]. Briefly, from each fetus six parallel sets were produced each spanning the entire body from eye to eye consisting of 24 cryosections, 25 μm thick and spaced 150 μm apart. The automated device for performing non-radioactive ISH using digoxigenin-labelled riboprobes on such sets has been previously described [9, 13, 14]. A total of 2961 transcripts were analyzed by our lab this way. The resulting micrographs were deposited on GenePaint and contributed to the EURExpress project. We used these data as well as the GenePaintEURExpress resources to annotate, 1667 genes for the eye, 1012 for teeth and 2000 randomly chosen genes for the ear and sensory system (present study).

For this present study, the full set of sections for a given probe was re-analyzed and observed under a light microscope (Leica DMLB). Imaging was performed with a CoolSNAP digital camera and corresponding software at different magnifications up to 0.2 μm/pixel, leading to the production of 11,000 micrographs for the 2000 randomly chosen genes.

### ImAnno computational infrastructure

**Fig 2:**
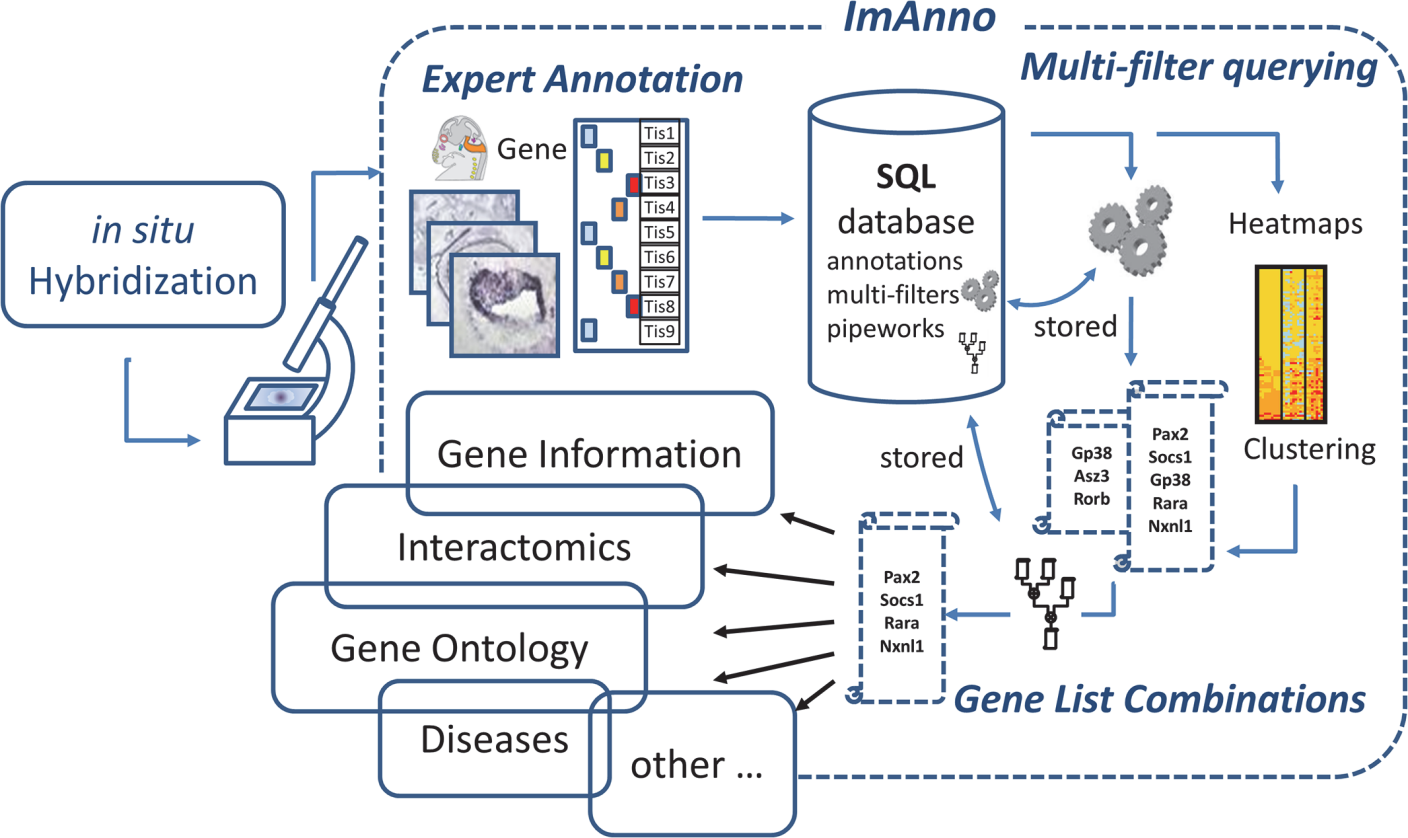
Overview of the ImAnno framework. The ISH images and annotations are integrated in a relational database. Filters and combinations of filters can be defined and stored by the user, providing gene lists for further integrated analysis with local and external databases (Gene information, Interactomics data, Gene Ontology…).

ImAnno is based on a relational database architecture (Fig. 2), and incorporates original annotation and filtering/analysis tools. The database *infrastructure* is designed to manage simultaneously multiple annotation projects performed by experts and the annotation, storage, access and analysis processes have been conceived for exploitation through a web user interface. New project and database features can be instanciated in the framework of collaboration.

To initiate a project, the project manager defines: i) the central object under study (in our case this is a gene, but it could be a patient, an image or a protein 3D structure, etc.), ii) the list of criteria associated with each object (in our case, the expression in 25 tissues, for a patient it could be a list of symptoms), iii) a scoring system with exclusive values for all defined criteria (e.g. negative, weak, medium, strong, not analyzed), iv) the source of the data (e.g. internet localization or filename of the ISH images). Once these four main items have been defined, the appropriate database schema is generated automatically and the annotation process can begin.

The input data can be either stored on the local system or accessible *via* internet, and an automated referencing in the database is performed once the data or its localization is integrated in the project. The list of criteria and scoring system can be modified throughout the duration of the project.

Within a project, in addition to the display facilities, ImAnno offers various querying and integrated analysis tools to search for objects or criteria using their associated annotations (see below).

**Annotation of ISH images.** The web-based interface for digital image annotation allows the authorized annotators to browse a set of ISH images associated with a given gene and to annotate the 25 studied tissues (labelled T1 to T25, Fig. 1 and Table 1). In accordance with the GenePaint annotation system [9], the tissue-specific expression level of a gene was assigned one of 5 values: negative, weak, medium, strong, not analyzed (Fig. 1B). Additional annotations can be made to describe the expression pattern, either selected from a predefined list of keywords (patchy, spotted, generalized) or as "free text". Any authorized user can annotate or re-annotate any tissue from any gene as long as the gene is not marked as "approved". The 11,000 images used and annotated in this project are stored in the ImAnno knowledgebase (imanno.lbgi.fr). As several images from different sections with different magnifications are often necessary to precisely analyze a gene expression pattern, users can also upload their own images or provide URLs to visualize images available on any digital atlas site, or any gene-related web site (Fig. 1A).

**Table 1:** Gene expression in the 25 tissues.

Ti	n.a.	%n.a.	neg.	%neg.	weak	%weak	medium	%medium	strong	%strong	express	%express	Ti.	Name
T1	6	0.3%	977	48.9%	685	34.2%	284	14.2%	48	2.4%	1017	50.9%	T1	External acoustic meatus
T2	28	1.4%	1248	62.4%	538	26.9%	166	8.3%	20	1.0%	724	36.2%	T2	Middle ear ossicles
T3	7	0.3%	1090	54.5%	749	37.5%	136	6.8%	18	0.9%	903	45.1%	T3	Middle ear tympanic membrane
T4	4	0.2%	868	43.4%	757	37.9%	305	15.2%	66	3.3%	1128	56.4%	T4	Middle ear mesenchyme
T5	10	0.5%	1277	63.9%	449	22.4%	205	10.2%	59	3.0%	713	35.6%	T5	Otic capsule
T6	8	0.4%	905	45.2%	752	37.6%	290	14.5%	45	2.2%	1087	54.4%	T6	Inner ear mesenchyme
T7	5	0.2%	892	44.6%	739	37.0%	288	14.4%	76	3.8%	1103	55.1%	T7	Stato-acoustic ganglion
T8	1	0.1%	1132	56.6%	664	33.2%	178	8.9%	25	1.2%	867	43.4%	T8	Inner spiral sulcus
T9	0	0.0%	1215	60.8%	614	30.7%	153	7.7%	18	0.9%	785	39.2%	T9	Kolliker organ
T10	3	0.1%	1477	73.8%	412	20.6%	92	4.6%	16	0.8%	520	26.0%	T10	Stria vascularis
T11	5	0.2%	1561	78.0%	360	18.0%	65	3.2%	9	0.5%	434	21.7%	T11	Outer spiral sulcus
T12	10	0.5%	1116	55.8%	661	33.0%	187	9.3%	26	1.3%	874	43.7%	T12	Saccule sensory region
T13	8	0.4%	1133	56.6%	671	33.5%	167	8.3%	21	1.1%	859	43.0%	T13	Saccule other cells
T14	10	0.5%	1114	55.7%	663	33.1%	188	9.4%	25	1.2%	876	43.8%	T14	Utricule sensory region
T15	8	0.4%	1131	56.5%	671	33.5%	166	8.3%	24	1.2%	861	43.0%	T15	Utricule other cells
T16	10	0.5%	1110	55.5%	662	33.1%	192	9.6%	26	1.3%	880	44.0%	T16	Crista sensory region
T17	9	0.5%	1132	56.6%	670	33.5%	164	8.2%	25	1.2%	859	43.0%	T17	Crista other cells
T18	11	0.6%	1112	55.6%	649	32.5%	198	9.9%	30	1.5%	877	43.9%	T18	Semi-circular canals
T19	35	1.8%	1386	69.3%	470	23.5%	97	4.8%	12	0.6%	579	28.9%	T19	Endolymphatic organ
T20	6	0.3%	1022	51.1%	651	32.5%	264	13.2%	57	2.9%	972	48.6%	T20	Choroide plexus 4th ventricle
T21	0	0.0%	455	22.8%	793	39.6%	570	28.5%	182	9.1%	1545	77.2%	T21	Hindbrain
T22	12	0.6%	824	41.2%	725	36.2%	378	18.9%	61	3.0%	1164	58.2%	T22	Sensory retina
T23	6	0.3%	818	40.9%	830	41.5%	299	14.9%	47	2.4%	1176	58.8%	T23	Olfactory organ
T24	11	0.6%	1216	60.8%	571	28.6%	153	7.7%	49	2.5%	773	38.6%	T24	Cartilage primordium of ribs
T25	14	0.7%	894	44.7%	685	34.2%	326	16.3%	81	4.0%	1092	54.6%	T25	Follicles of vibrissae

Gene numbers and percentages ranked according to their expression status (negative, weak, medium, strong) in the 25 tissues. n.a.: not analyzed (i.e. ISH signal in a given tissue that was impossible to determine reliably).

**Data management, querying and visualization tools.** Information about the annotators, the history of the annotation process, the genes, their corresponding images, the tissue-specific expression data as well as the free text comments or links to external sites are also stored in the ImAnno knowledgebase and can be queried through the ImAnno web-based interface. The web site includes a user authentication system with appropriate read/write access rights (public, private and user sub-group) defined by the project manager to allow different levels of processing, querying and visualization of the database.

The data in ImAnno can be queried using the gene name, nucleotide or protein sequence (Blast search) or any information associated with the genes, in order to access the complete set of images (Fig. 1F) and annotations together with cartoon-like schemes showing all the analyzed tissues colored according to the expression level (blue: negative, yellow: weak, orange: medium, red: strong) (Fig. 1 B-E). In addition, ImAnno provides a computational filter (called “Sieve” on the web interface) to retrieve lists of genes according to their tissue-specific expression patterns. This filter tool allows the user to define a Boolean combination ("AND", "OR") of the annotations, which can then be saved for future pattern searches (S1 Fig.). Furthermore, lists of genes can be combined using logical union, intersection and/or complement operations *via* a dynamic HTML form to perform further functional analysis.

**Fig 3:**
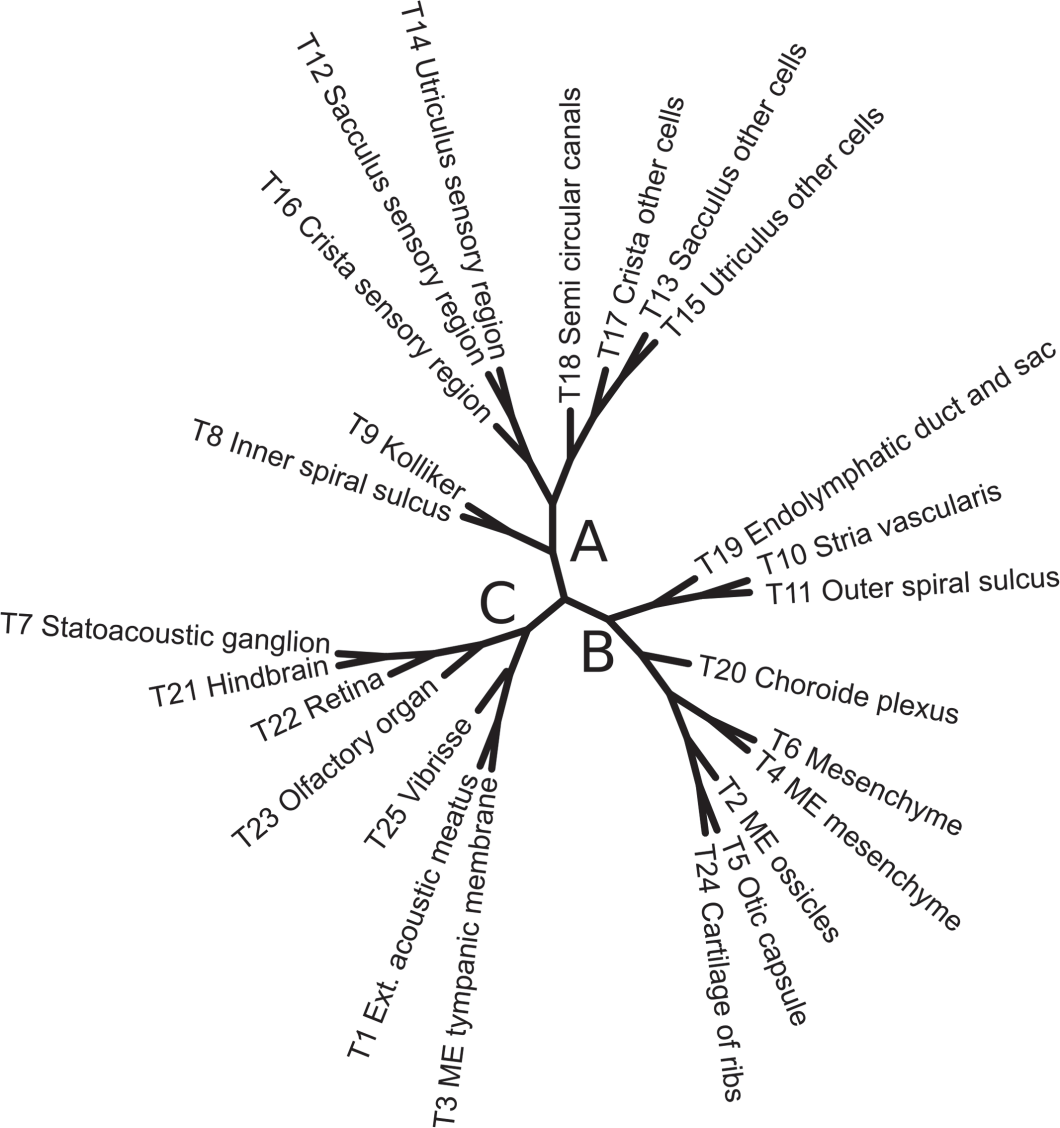
Dendrogram of tissue expression. The three main branches (A, B and C) are described in the main text. IE, inner ear; ME, middle ear.

**Functional genomics analysis.** ImAnno offers additional tools to characterize gene lists related to:

- Interactomics data obtained from the STRING database [15], containing known or predicted physical and functional protein-protein interactions. Only interactions with high confidence levels (>0.7) are used, as suggested by the authors. Interactomic networks can be visualized using CytoscapeWeb [16] or downloaded (networks and colors) for input to the Cytoscape software [17]. Starting with a gene set provided by the user (the "query genes"), the StringInteractome module provides three different "zooming levels": level 1, the standard output displaying the query genes and all the genes exhibiting at least one interaction with the query genes; level 2, the intermediate level (obtained after filtering the level 1 list) displays the query genes and the genes interacting with at least two query genes; level 3, the "Query network", displaying only direct interactions between query genes.
- Relationships with human disorders with Mendelian inheritance, based on the morbidmap data from the OMIM database (http://www.omim.org).
- Gene ontology (version 9.05) (http://www.geneontology.org). For a gene list, attached GO terms can be displayed. For two gene lists A and B, GO terms can be ordered according to the fold enrichment determined as the percentage of genes from A verifying a given GO term over the percentage of genes from B verifying the same GO term. This fold enrichment can be used to evaluate an over- or under- representation of GO term. In the present study, we use molecular function as the default term.
- Dendrogram of tissue correlation. A distance matrix based on the expression values of the 2000 annotated genes in the 25 tissues was computed using a Spearman’s rank correlation coefficient with the following numeric values: 0: negative, 2: weak, 3: medium and 4: strong. This distance matrix was input to the FastME program [18] in order to construct a tissue expression dendrogram (Fig. 3). Clustering with the Secator program [19] is in agreement with the dendrogram. The dendrogram can be annotated and colored using the DecoreTree module directly on the web interface.

## Results and Discussion

The ImAnno infrastructure provides a centralized, structured knowledgebase infrastructure for storing very precise information about the expression of genes in anatomically detailed tissues corresponding to the eye, the teeth and the ear and sensory organs. The knowledgebase is accessible *via* a user-friendly web interface, allowing multiple expert annotators to share project data within a framework defined according to the anatomical properties of the studied organ. As illustrated below, besides the annotation process, the ImAnno interface was used to perform integrated analyses combining the experimental expression data with other knowledge.

### ISH expression patterns in 25 tissues from the ear and sensory systems

Among the 2000 mouse genes analyzed, 16.5% (331) did not exhibit an ISH signal in any of the 25 tissues investigated. This value is comparable to the 18% of genes with no expression in the whole embryo, as reported in the EURExpress database [5]. The majority of the remaining transcripts exhibited a weak signal in the 25 tissues (Table 1), which is not surprising since previous analyses showed that most transcripts (86%) were expressed by less than five copies per cell [20]. More than half of the genes were expressed in the hindbrain and sensory organs (notably the sensory retina, olfactory epithelium and vibrissae follicles). For most of the inner ear tissues, less than 50% of the gene transcripts were "expressed".

To further characterize the expression profiles of the 2000 annotated genes, we calculated a tissue dendrogram of the ISH expression levels observed in the 25 tissues analyzed (Fig. 3). Three main branches were observed, corresponding to tissues that share some common functions and/or embryonic origin. Branch A (tissues T12 to T18) corresponds to inner ear tissues with both functional and embryonic relationships since they are mainly of ectodermal origin. Among the utricle, saccule and crista tissues, the cells involved in sensory functions (T12, T14, T16) clearly branch separately from the other adjacent cell types (T13, T15, T17). Branch B includes structures derived from a common embryonic mesodermal origin, but it is more heterogeneous from a functional point of view. Nevertheless, functional fate is also visible in this branch, since the three future bony or cartilage structures (T2, T5, T24) appear closely related, despite their very different anatomical localization (middle ear, inner ear and ribs). Branch C is composed of two groups of tissues. The first group includes the nervous tissues (T7,T21–23), which are functionally close despite different embryological origins [21]. This functional proximity may explain their close relationships with respect to expression of genes controlling neural differentiation [14]. The second group, functionally diverse but composed of skin derived organs (T1, T3, T25), includes tissues mainly of ectodermal origin such as the vibrissae follicles and the external acoustic meatus, formed from the ectoderm of the first branchial cleft, and the tympanic membrane made of a fibrous layer of mesodermal origin surrounded by two epithelial layers [22].

### Analysis of genes expressed in 5 developing sensory organs

We analyzed the expressed genes in the five ‘KUROV’ developing sensory organs (i.e. K: Kölliker’s organ, U: Utricle sensory region, R: Retina, O: Olfactory organ and V: Vibrissae follicles), all potentially responsible for the transduction of external information into internal signals, focusing our investigation on the sensory epithelia. From an embryological point of view, these five ‘KUROV’ tissues are diverse since K, U and O are derived from sensory placodes, while V originates from the placodes of the muzzle’s skin and R from the neuroepithelium.

**Fig 4:**
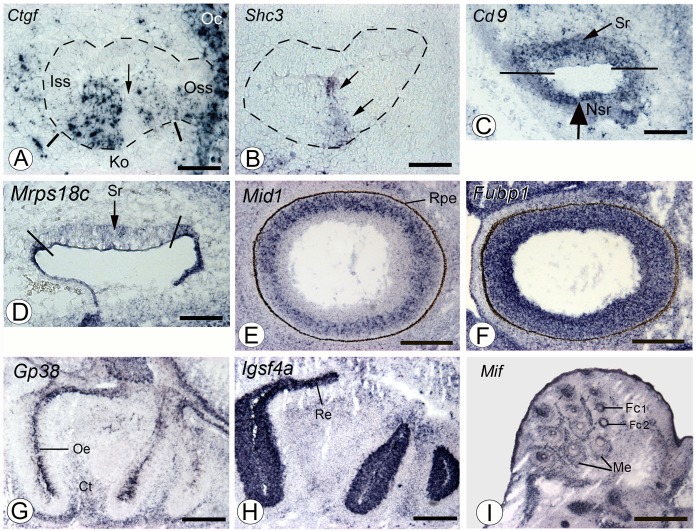
ISH images illustrating gene expression patterns in sensory organs. Gene names are indicated in the upper left part of the image. A,B: Kölliker organ (Ko). The basal cochlear canal is delineated by dashed lines. Ko is found in the ventral region (delineated by two bars). Patchy expression of Ctgf (Connective tissue growth factor) restricted to Ko extending toward the outer spiral sulcus (Oss) and the otic capsule (Oc) and absent in the inner spiral sulcus (Iss). The arrow highlights a region devoid of Ctgf expression in Ko. This latter region expresses Shc3 (Src homology 2 domain-containing transforming protein C3) (arrows in B). Scale bar: 50 μm. C,D: Utricle. Cd9 (Cd9 antigen) is expressed both in the prospective sensory (Sr, arrow) and non-sensory (Nsr, large arrow) regions. Lines: separation between Sr and Nsr. Mrps18c (mitochondrial ribosomal protein S18C) is weakly expressed in Sr and strongly in NSr. Scale bar: 100 μm. E,F: Retinal expression of Mid1 (Midline 1) and Fubp1 (Far upstream element [FUSE] binding protein 1). Rpe: retinal pigmented epithelium. Scale bar: 200 μm. G,H: Olfactory organ. Gp38 (Glycoprotein 38) is expressed in the olfactory epithelium (Oe) and the cartilage primordia of turbinate bones (Ct). Igsf4a (Immunoglobulin superfamily, member 4A) is strongly expressed throughout the olfactory and respiratory (Re) epithelia. Scale bar: 200 μm. I: Expression of Mif (Macrophage migration inhibitory factor) in the primordia of vibrissae follicles. Fc1, Fc2: different regions of the follicle cells distinguished by section. Me: mesenchyme surrounding the vibrissae. Scale bar: 500 μm.

Fig. 4 illustrates some archetypal expression patterns observed in the individual sensory tissues and the gene expression behavior in each individual sensory tissue is provided in S1 Table.

**Cochlea sensory organ or the Kölliker’s organ (K).** Of the 2000 annotated genes, 785 (39.2%) showed an ISH signal in this structure and only 9 genes had no expression in any of the other four sensory tissues (named ‘absent urov’ in S1 Table). The expression patterns observed in Kölliker’s organ are diverse, with some genes exhibiting an expression throughout the whole organ (e.g. Ctgf in Fig. 4A) or a very restricted pattern (e.g. Shc3 in Fig. 4B).

**Utricular sensory region (U).** 876 genes (43.8%) showed an ISH signal in the prospective vestibular receptors, their supporting cells, and/or some neighboring cells such as transitional cells. Several genes showed a particularly strong expression in the utricular epithelium (e.g. Cd9 and Mrps18c, Fig. 4C and D).

**Sensory retina (R).** 1164 transcripts (58.2%) were expressed in the epithelium of the neural retina. Of these genes, 36.2% exhibited a weak expression and 61 (3.0%) of the expressed transcripts showed a strong signal (S1 Table, Fig. 4E and F).

**Olfactory organ (O).** ISH signals were observed in the olfactory organ for 1176 (58.8%) transcripts. Some genes were differently expressed, such as Gp38 which is strongly expressed but restricted to the olfactory epithelium or Igsf4a present in the whole organ (Fig. 4G and H).

**Vibrissae follicles (V).** 1092 (54.6%) transcripts showed an ISH signal in the vibrissae follicles. Some genes showed strong expression (e.g. Mif, Fig. 4I) and many of their human orthologs were found to be expressed in a microarray study of human head hair follicles [23].

### Functional network integrated analysis

We then investigated the gene functions, interaction patterns and human pathologies related to the 623 genes expressed concomitantly in all five sensory epithelia (hereafter termed the KUROV genes listed in S2 Table).

**Fig 5:**
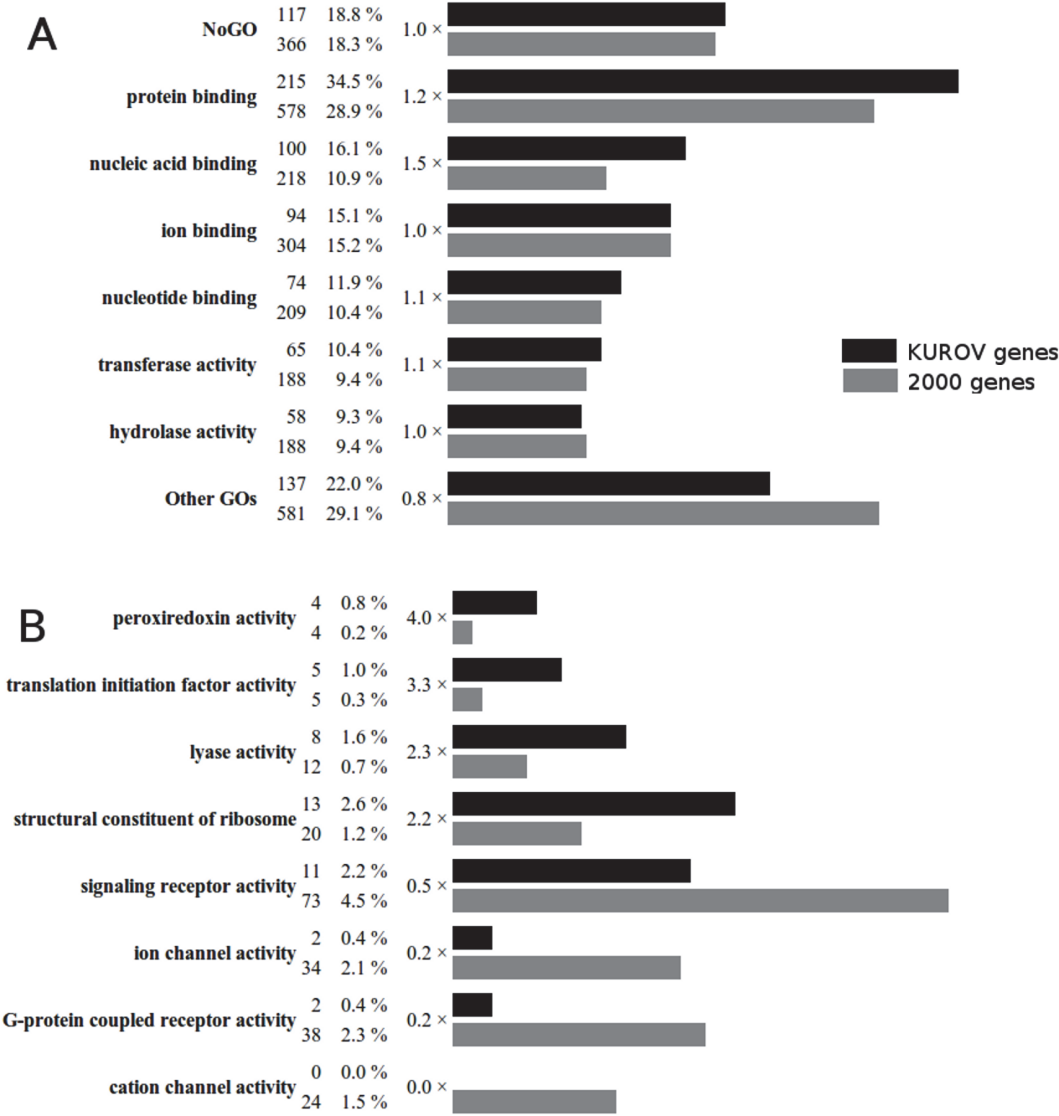
Comparison of the ‘Molecular Function’ GO term distributions for the 623 KUROV (black) and 2000 (gray) genes. A: Most populated GO terms. After each GO term, the total number of genes verifying a given GO term among the 623 KUROV genes and the 2000 annotated genes are indicated followed by their corresponding percentages and the ratio of these percentages. For sake of clarity, only GO terms present in more than 7% of the respective gene sets are shown. NoGO: genes with no assigned GO term. Other GOs: sum of all other categories. B: Biologically relevant GO terms exhibiting over- or under-representation using the highest and lowest percentage ratio.

**Gene functions.** We first verified that the overall distribution of the gene ontology (GO) terms observed for the complete set of 2000 genes is comparable to the distribution observed for the complete non redundant mouse proteome (data not shown). This supports our hypothesis that these randomly chosen genes are representative of the mouse proteome. We then compared the GO annotations of the 2000 genes and the 623 KUROV genes. Of the 2000 genes, 366 (18.3%) have no GO annotation, almost the same percentage as for the KUROV group (117 genes, 18.8%). The most significant GO terms (representing at least 40 genes) are shown in Fig. 5A. By comparing the two gene sets we observed a major difference linked to the lower proportion of the “other” GO terms and the higher proportion of “protein binding” and “nucleic acid binding” GO terms observed in the KUROV group. This may indicate some enrichment in the KUROV genes for specialized functions related to these two binding functions.

Detailed analysis of the relative distributions of GO terms in the 2000 and KUROV genes respectively (Fig. 5B) reveals an over-representation of housekeeping genes, such as those with “translation initiation factor activity” or “structural constituent of ribosome” annotations, as well as a drastic under-representation of the “ion channel” and “G-protein coupled receptor” (GPCR) activities. Such under-representation of channel and GPCR activities in sensory organs might appear paradoxical, but it should be noted that the KUROV group includes only genes co-expressed in the 5 sensory tissues. Thus, this under-representation may reflect the fact that these activities are related to highly specialized cellular functions, which are probably specific to each or some sensory organs. Another plausible hypothesis is that these sensory organs have not yet reached their functional status and thus do not express the complete set of ion channel and GPCR activities at this period of development [24,25,26,27]

**Fig 6:**
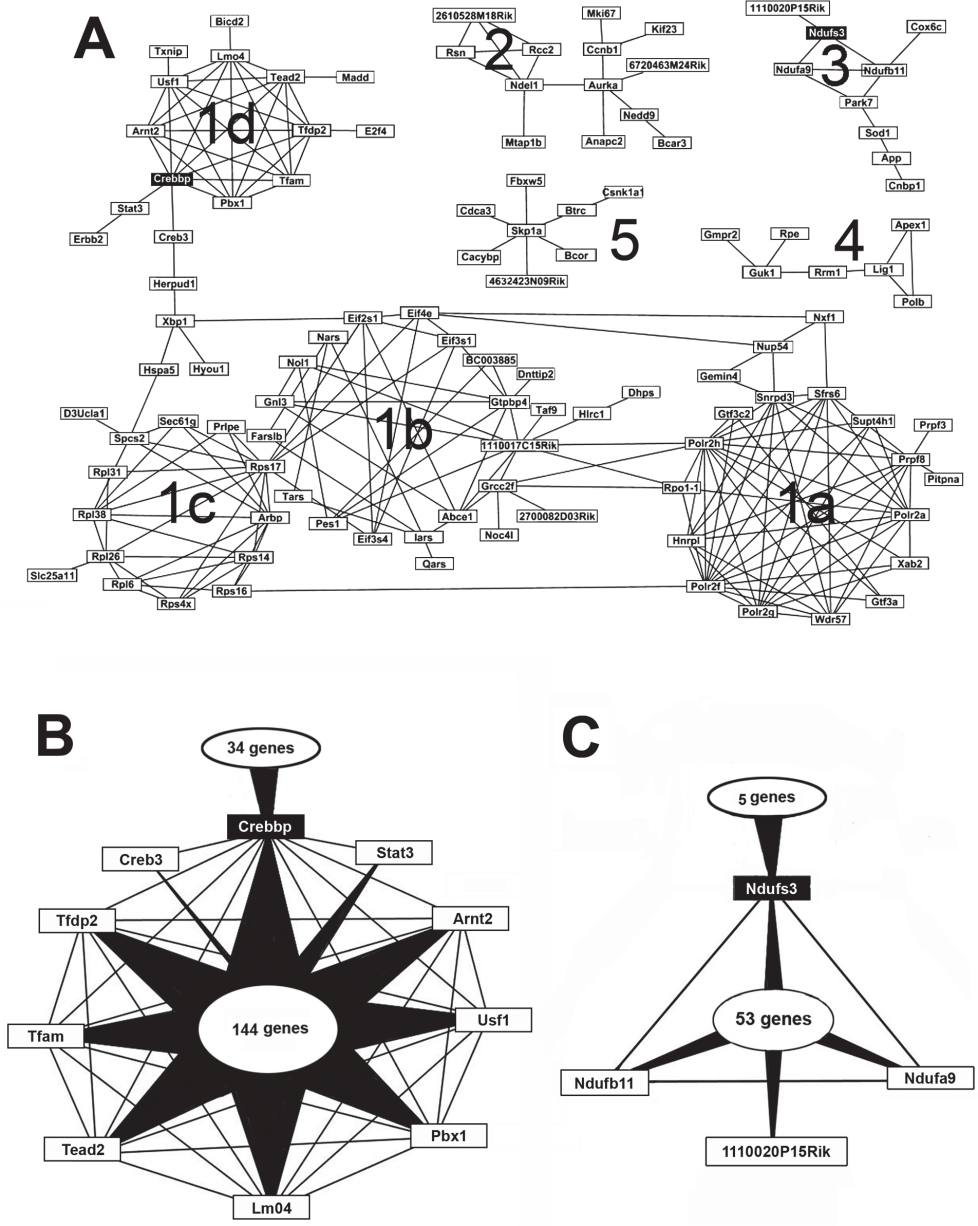
Interactome networks for the KUROV genes. A: Networks (gene names boxed) with direct interactions between KUROV genes (level 3). The largest network (1) with sub-networks (1a to 1d) and less populated networks are shown (2 to 5). Black boxes: gene interactions examined in B and C. B: Schematic representation of level 2 interaction network of Creppb (CREB-binding protein) depicting the 144 non-KUROV genes joining the 9 KUROV genes from sub-network 1d. C: Level 2 interaction network of Ndufs3 (NADH-Ubiquinone oxidoreductase Fe-S protein 3) with the 53 non-KUROV genes joining the 3 KUROV genes (Ndufb11, Ndufa9 and 1110020P15Rik) from network 3.

**Network analysis.** We then investigated the interaction patterns present in the gene lists using various "zooming levels" (see Material and Methods). Among the 623 KUROV genes, 168 have direct interactions (level 3) with other KUROV genes. Fig. 6A shows 112 of them distributed in one principal network and 4 small ones. The largest network is composed of 75 genes distributed in 4 sub-networks (1a, 1b, 1c and 1d), while the smaller networks (2–5) are composed of 13, 9, 7 and 8 genes respectively. As previously noted, these interaction networks shared by the five sensory organs are mainly composed of housekeeping genes related to processing of genetic information (transcription, translation, RNA transport, ribosome synthesis…). For example, the sub-network 1a is composed of several RNA polymerases (*Polrs*), spliceosome genes (*Wdr57, Xab2*) and genes involved in nuclear ribonucleoprotein synthesis (*Hnrpl, Snrpd3*). The sub-network 1b mainly contains genes involved in tRNA synthesis (*Nars, Fars1b, Iars, Tars*) and transport (*Eif* genes). The *Rps17* (Ribosomal protein S17) connects the sub-networks 1b and 1c, the latter mainly including cytoplasmic and mitochondrial ribosomal proteins. Nevertheless, these sub-networks can be connected *via* multiple genes *(Nxf1* and *Nup54* toward *Eif4e*; *Polr2h, Rpo1-1* toward *1110017c15Rik, Grcc2f; Polr2f* to *Rps16*), suggesting various functional relationships and common pathways. In the sub-network 1d, most genes encode transcription factors (*Tfam, Tfdp2, Usf1*), while *Crebbp* is involved in Wnt, cell cycle and Notch signaling pathways. It is connected to the sub-network 1c through several genes involved in protein processing in endoplasmic reticulum (*Herpud1, Xbp1*). The network 2 is mainly involved in cellular processes like cell cycle with several genes (*Rcc2, Ccnb1, Aurka, Anapc2, Kif23*), while several NADH dehydrogenases are involved in metabolic pathways such as oxydative phosphorylation (*Nduf* genes present in network 3). Most genes of network 4 are implicated in nucleotide metabolism, while the network 5 is enriched in signaling pathways and cellular processes such as Wnt, Hedgehog, Hippo, TGF-β signaling pathways and adherens junctions.

By mining the 623 KUROV genes *via* the "zooming levels" 1 and 2, we identified 377 KUROV genes that are related to 3277 genes *via* a single connection (level 1), among which 324 KUROV genes are connected *via* 1653 "intermediate" genes (data not shown). We focused on two genes: *Crebbp* (encoding CREB-binding protein) from the sub-network 1d and *Ndufs3* (encoding NADH-ubiquinone oxidoreductase Fe-S protein 3) from the network 3 (black-boxed in Fig. 6A). Fig. 6B displays a schematic representation of the level 2 interactions of the *Crebbp* gene. This gene has direct interactions with 9 KUROV genes (*Stat3, Arnt2, Usf1, Pbx1, Lmo4, Tead2, Tfam, Tfdb2, Creb3*), thus defining 144 genes connecting at least 2 of the 10 KUROV genes. Finally, 34 additional genes connect *Crebbp* to other KUROV genes, not represented here. At the functional level, these genes exhibit a strong enrichment in nucleic acid binding functions and more precisely, in transcription factors (133 out of 188), most of them being involved in developmental processes and response to growth factors. *Ndufs3*, the second example illustrated in Fig. 6C, is involved in oxidative phosphorylation and directly connected with 8 KUROV genes, among which 3 genes (*Ndufb11, Ndufa9* and *1110020P15Rik)* share 53 genes with *Ndufs3 via* a level 2 type interaction. This gene set exhibits striking enrichment in specific functions since 33 of the 53 genes belong to the Nduf family, while 10 of the 20 non-Nduf genes correspond to hydrogen ion transmembrane transporters. The Nduf family is involved in several brain diseases such as Alzheimer, Parkinson and Huntington diseases. Several of these genes, such as *Ndufs3* and *Ndufa9*, are also involved in the Leigh syndrome due to mitochondrial complex I deficiency (http://omim.org/entry/256000), although the genetic origin of this syndrome presents a large heterogeneity (Di Mauro & De Vivo, 1996).

**Fig 7:**
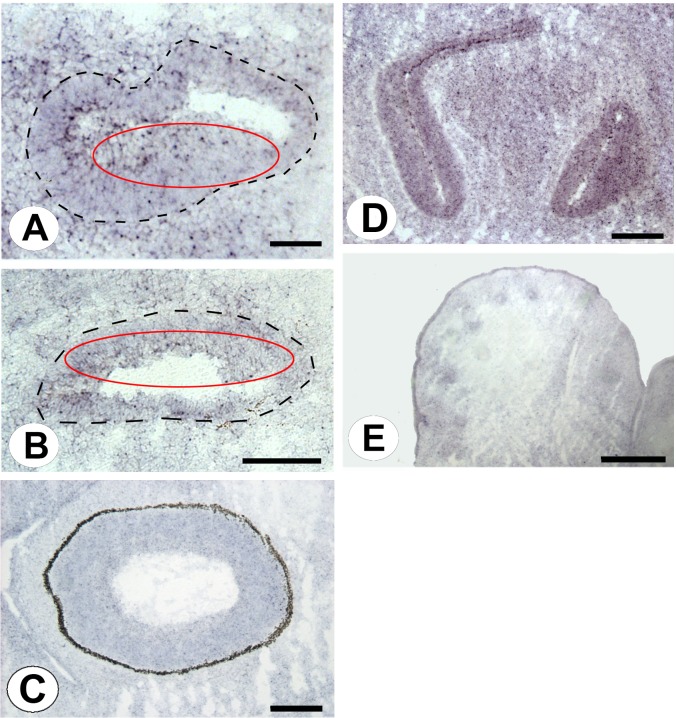
Crebbp expression in the five sensory organs. Kölliker's organ (A), Utricle (B), Retina (C), Olfactory organ (D) and Vibrissae (E). The dashed lines delineate the basal cochlear canal containing Kölliker's organ (A) and the utricle (B). Red ellipses identify the sensory regions. Scale bars: A, 50 μm; B, 100 μm; C, 200 μm; D, 200 μm; E, 500 μm.

**Human diseases.** To allow the user to associate the genes with potential human diseases, ImAnno offers the possibility to directly query the Morbid Map Database from OMIM implemented in our infrastructure. As an example, we focused on the 623 KUROV genes and found that 74 are known to be related to diseases (S3 Table) with 15 corresponding to specific syndromes related to impairment of some sensory functions, such as *Aldh3a2* for Sjogren-Larsson syndrome, *Hsp4* for Hermansky-Pudlak syndrome 4, *Slc12a1* for Bartter syndrome (type 1) and *Crebbp* for Rubinstein-Taybi syndrome. The latter syndrome affects several sensory organs and is characterized by hearing loss, hirsutism, glaucoma and nose malformations as well as mental retardation [28]. *Crebbp* was found to be weakly expressed in the five developing sensory organs (Fig. 7). These complex interactions are probably related to functional processes affecting the development or the function of sensory organs. This is not surprising since *Crebbp* is involved in several basic cellular pathways operating during development and cell cycle, and may thus be involved in specific diseases such as DiGeorge syndrome, ovarian tumorigenesis, prostate cancer [29,30,31] and cognitive functions like long-term memory formation [32]. The *Lmo4* gene is related to *Crebbp*, (not found in the Morbid Map) and is remarkable in that its targeted disruption is lethal at birth, affecting the inner ear and the retina [33,34]. Based on our observations of the ISH data, we predict that *Lmo4* may also be important for the development of vibrissae follicles, and possibly for all hair follicles. Moreover, it may be essential for cochlear function as well as to the olfactory organ.

## Conclusions

Initially designed to facilitate the detailed and integrated analysis of complex organs, notably the ear, ImAnno is now being used for other large scale annotation projects for instance, in the developing mouse eye and teeth or the human eye fundus project (imanno.lbgi.fr). This demonstrates the usefulness and ergonomy of the knowledgebase infrastructure, which can combine object-centric, multi-level annotation (gene, patient, etc.) and analysis of image data through various data mining tools of GO annotation, interactomics or human disease features.

As a proof of concept, using the expert annotation of ISH gene expression of 2000 randomly chosen genes, we focused our ImAnno study on the E14.5 developmental stage using 25 annotated embryonic tissues with special emphasis on 5 developing sensory tissues (KUROV). Hierarchical clustering demonstrated that expression patterns can be used to identify tissues with a common embryological origin, such as ectoderm or mesoderm derived tissues, or with similar potential functions such as nervous tissues. Thus, we infer that, at the E14.5 stage, embryological origin is no longer the only source of expression pattern enrichment and that some genetic pathways important for the onset of sensory function are already present in some tissues. Surprisingly, a comparison of the functional enrichments observed in the 2000 genes and in the sensory gene set revealed that "sensory" functions are drastically under-represented in the sensory gene set, suggesting that, at E14.5, the 5 developing sensory tissues may share a limited number of sensory genes or may have not yet reached their functional status. The incomplete functional status of the sensory tissues is further suggested by mining of the interactomics data, which highlights gene pathways including mainly housekeeping genes and signaling pathways (Wnt, Notch, Jak-STAT, Hedgehog). Taken together, these data strongly support the hypothesis that, for sensory tissues, E14.5 is a pivotal stage between the embryonic-developmental stage and the beginning of the fully functional phase that will be reached for many sensory organs only after birth.

## Supporting Information

S1 Fig Computational filter for Boolean tissue expression querying. (TIF)

S1 Table Gene expression behavior in each individual sensory tissue. The numbers (nbGenes), percentages (%) and names (gene list) of genes expressed in each of the five developing sensory organs (Kölliker's organ, Utricule, Retina, Olfactory organ and Vibrissae). Percentages are relative to the 2000 annotated genes. Gray lines indicate genes expressed in one specific tissue and absent in the four other tissues. The lines “w+m+s” give the total numbers of genes exhibiting weak, medium or strong expression in each organ.
(XLSX)

S2 Table List of the 623 genes co-expressed in KUROV organs. (DOCX)

S3 Table List of the 74 KUROV genes related to diseases according to the Morbid Map database. (XLSX)

## References

[1] Christiansen JH, Yang Y, Venkataraman S, Richardson L, Stevenson P, et al (2006) EMAGE: a spatial database of gene expression patterns during mouse embryo development. Nucleic Acids Res 34, D637–D641. 16381949 10.1093/nar/gkj006 PMC1347369

[2] Stansberg C, Vik-Mo AO, Holdhus R, Breilid H, Srebro B, et al (2007) Gene expression profiles in rat brain disclose CNS signature genes and regional patterns of functional specialisation.BMC Genomics Apr 4, 8:94 17408481 10.1186/1471-2164-8-94 PMC1853090

[3] Smith CM, Finger JH, Hayamizu TF, McCright IJ, Eppig JT, et al (2007) The mouse Gene Expression Database (GXD): 2007 update. Nucleic Acids Res. Nucleic Acids Res 35, D618–D623. 17130151 10.1093/nar/gkl1003 PMC1716716

[4] Barrett T, Troup DB, Wilhite SE, Ledoux P, Rudnev D, et al (2009) NCBI GEO: archive for high-throughput functional genomic data. Nucleic Acids Res 37, D885–D890. 10.1093/nar/gkn764 18940857 PMC2686538

[5] Diez-Roux G, Banfi S, Sultan M, Geffers L, Anand S, et al (2011) A high-resolution anatomical atlas of the transcriptome in the mouse embryo. PLoS Biol 9, e1000582 10.1371/journal.pbio.1000582 21267068 PMC3022534

[6] Maienschein-Cline M, Zhou J, White KP, Sciammas R, Dinner AR (2012) Discovering transcription factor regulatory targets using gene expression and binding data. Bioinformatics 28, 206–213. 10.1093/bioinformatics/btr628 22084256 PMC3259433

[7] Geffers L, Herrmann B, Eichele G (2012) Web-based digital gene expression atlases for the mouse. Mamm Genome 23, 525–358. 10.1007/s00335-012-9413-3 22936000

[8] Rustici G, Kolesnikov N, Brandizi M, Burdett T, Dylag M, et al (2013) ArrayExpress update-trends in database growth and links to data analysis tools. Nucleic Acids Res 41, D9879–9890. 10.1093/nar/gks1174 PMC3531147 23193272

[9] Visel A, Thaller C, Eichele G (2004) GenePaint.org: an atlas of gene expression patterns in the mouse embryo. Nucleic Acids Res 32, D552–556. 14681479 10.1093/nar/gkh029 PMC308763

[10] Waterston RH, Lindblad-Toh K, Birney E, Rogers J, Abril JF, et al (2002) Initial sequencing and comparative analysis of the mouse genome. Mouse Genome Sequencing Consortium. Nature 420, 520–562. 12466850 10.1038/nature01262

[11] Lindsay S, Copp AJ (2005) MRC-Wellcome Trust Human Developmental Biology Resource: enabling studies of human developmental gene expression. Trends Genet 21, 586–590. 16154230 10.1016/j.tig.2005.08.011

[12] Eichele G, Diez-Roux G (2011) High-throughput analysis of gene expression on tissue sections by *in situ* hybridization. Methods 53, 417–423. 10.1016/j.ymeth.2010.12.020 21185383

[13] Carson JP, Thaller C, Eichele G (2002) A transcriptome atlas of the mouse brain at cellular resolution. Curr Opin Neurobiol 12, 562–265. 12367636 10.1016/s0959-4388(02)00356-2

[14] Visel A, Carson J, Oldekamp J, Warnecke M, Jakubcakova V, et al (2007) Regulatory pathway analysis by high-throughput in situ hybridization. PLoS Genet 3, 1867–1883. 17953485 10.1371/journal.pgen.0030178 PMC2041993

[15] Szklarczyk D, Franceschini A, Kuhn M, Simonovic M, Roth A, et al (2011) The STRING database in 2011: functional interaction networks of proteins, globally integrated and scored. Nucleic Acids Res 39, D561–568. 10.1093/nar/gkq973 21045058 PMC3013807

[16] Lopes CT, Franz M, Kazi F, Donaldson SL, Morris Q, et al (2010) Cytoscape Web: an interactive web-based network browser. Bioinformatics 26, 2347–2348. 10.1093/bioinformatics/btq430 20656902 PMC2935447

[17] Shannon P, Markiel A, Ozier O, Baliga NS, Wang JT, et al (2003) Cytoscape: a software environment for integrated models of biomolecular interaction networks. Genome Res 13, 2498–2504. 14597658 10.1101/gr.1239303 PMC403769

[18] Desper R, Gascuel O (2006) Getting a tree fast: Neighbor Joining, FastME, and distance-based methods. Curr Protoc Bioinformatics 6, Unit 6.3. 10.1002/0471250953.bi0603s15 18428768

[19] Wicker N, Perrin GR, Thierry JC, Poch O (2001) Secator: a program for inferring protein subfamilies from genetic trees. Mol Biol Evol 18, 1435–1441. 11470834 10.1093/oxfordjournals.molbev.a003929

[20] Zhang L, Zhou W, Velculescu VE, Kern SE, Hruban RH, et al (1997) Gene expression profiles in normal and cancer cells. Science 276, 1268–1272. 9157888 10.1126/science.276.5316.1268

[21] Rossant J, Tam PP (2002) Mouse development Patterning, Morphogenesis, and Organogenesis. Academic Press, San Diego.

[22] Mallo M (2003) Formation of the outer and middle ear, molecular mechanisms In: Development of Auditory and Vestibular Systems-3 Molecular Development of the Inner Ear Eds R Romand & I Varela-Nieto Elsevier/Academic Press, pp 85–113. 10.1016/s0070-2153(03)57003-x 14674478

[23] Kim SJ, Dix DJ, Thompson KE, Murrell RN, Schmid JE, et al (2006) Gene expression in head hair follicles plucked from men and women. Ann Clin Lab Sci 36, 115–126. 16682506

[24] Romand R, Despres G, Giry N (1987) Factors affecting the onset of inner ear function. Hear Res 28, 1–7. 3301775 10.1016/0378-5955(87)90148-1

[25] Blackshaw S, Harpavat S, Trimarchi J, Cai L, Huang H, et al (2004) Genomic analysis of mouse retinal development. PLoS Biol E247 15226823 10.1371/journal.pbio.0020247 PMC439783

[26] Tian H, Ma M (2008) Differential development of odorant receptor expression patterns in the olfactory epithelium: a quantitative analysis in the mouse septal organ.Dev Neurobiol 68, 476–86. 10.1002/dneu.20612 18214836 PMC2266684

[27] Nickell MD, Breheny P, Stromberg AJ, McClintock TS (2012) Genomics of Mature and Immature Olfactory Sensory Neurons. J. Comp Neurol 520, 2608–2629. 10.1002/cne.23052 22252456 PMC4023872

[28] Roelfsema JH, Peters DJ (2007) Rubinstein-Taybi syndrome: clinical and molecular overview. Expert Rev Mol Med 9, 1–16. 10.1017/S1462399407000415 17942008

[29] Wurdak H, Ittner LM, Lang KS, Leveen P, Suter U, et al (2005) Inactivation of TGF-beta signaling in neural crest stem cells leads to multiple defects reminiscent of DiGeorge syndrome. Genes Dev 19, 530–535, 15741317 10.1101/gad.317405 PMC551573

[30] Boyer A, Goff AK, Boerboom D (2010) WNT signaling in ovarian follicle biology and tumorigenesis. Trends Endocrinol Metab 21, 25–32. 10.1016/j.tem.2009.08.005 19875303

[31] Liu X, Li CH, Huang G, Ding C, Liu H (2012) Correlation analysis of JAK-STAT pathway components on prognosis of patients with prostate cancer. Pathol Oncol Res 18, 17–23. 10.1007/s12253-011-9410-y 21681602

[32] Maguschak KA, Ressler KJ (2010) Wnt signaling in amygdala-dependent learning and memory. J Neurosci 31, 13057–13067. 10.1523/JNEUROSCI.3248-11.2011 PMC3184457 21917789

[33] Duquette PM, Zhou X, Yap NL, MacLaren EJ, Lu JJ, et al (2010) Loss of LMO4 in the retina leads to reduction of GABAergic amacrine cells and functional deficits. PLoS One 5, e13232 10.1371/journal.pone.0013232 20949055 PMC2951357

[34] Deng M, Pan L, Xie X, Gan L (2010) Requirement for Lmo4 in the vestibular morphogenesis of mouse inner ear. Dev Biol 338, 38–49. 10.1016/j.ydbio.2009.11.003 19913004 PMC2812651

