# Supplementary figures and images for: Integrated Annotation and Analysis of In Situ Hybridization Images Using the ImAnno System: Application to the Ear and Sensory Organs of the Fetal Mouse
Source: PLoS One. 2015 Feb 23;10(2):e0118024. doi: 10.1371/journal.pone.0118024 (PMC4338146; doi:10.1371/journal.pone.0118024)

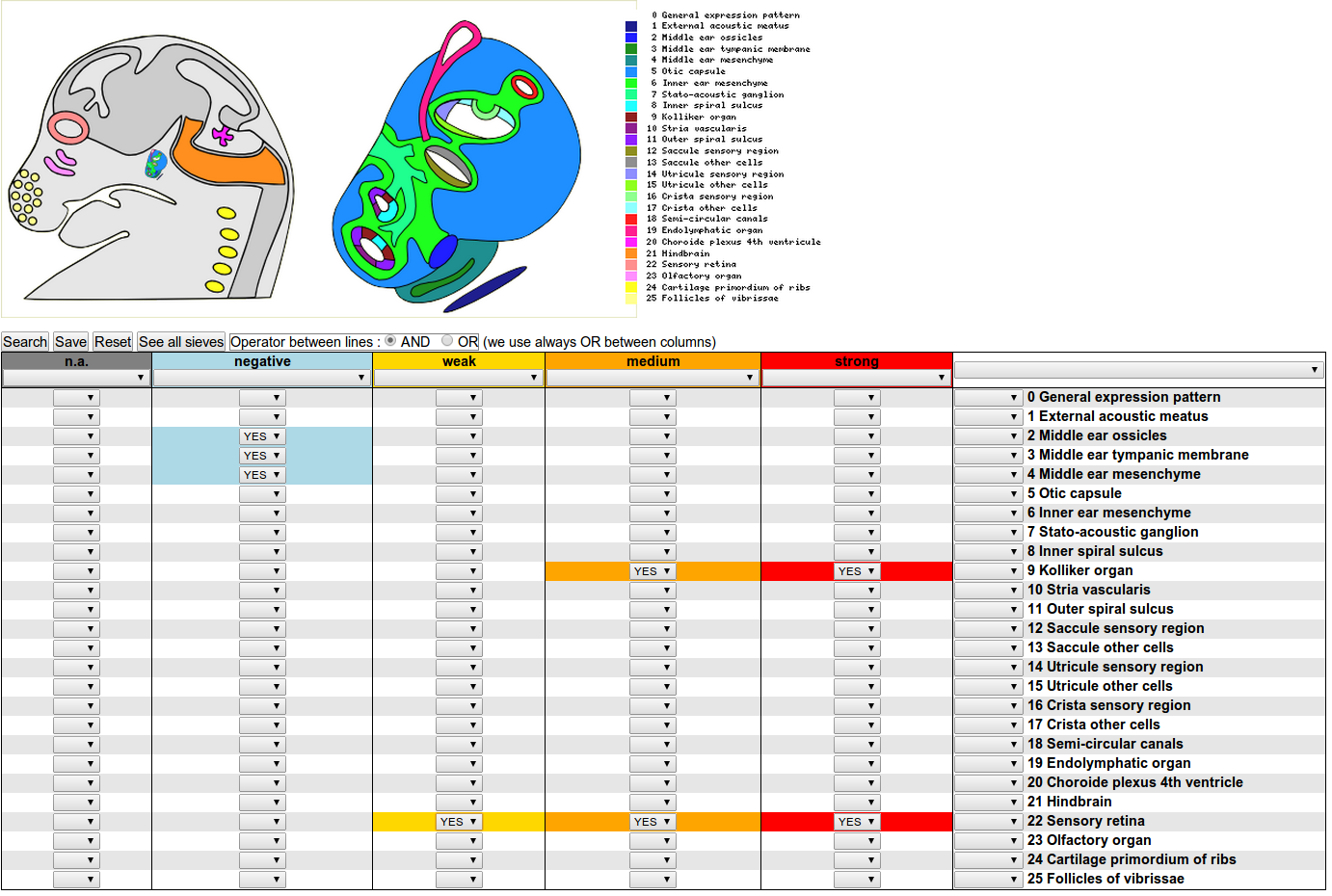

Supplement: S1 Fig — (TIF) [file pone.0118024.s001.tif]
