# Supplementary material for: Integrated Annotation and Analysis of In Situ Hybridization Images Using the ImAnno System: Application to the Ear and Sensory Organs of the Fetal Mouse
Source: PLoS One. 2015 Feb 23;10(2):e0118024. doi: 10.1371/journal.pone.0118024 (PMC4338146; doi:10.1371/journal.pone.0118024)
Supplement: S2 Table — (DOCX) [file pone.0118024.s003.docx]

**Table S2**

**List of genes of KUROV (623 genes present in the 5 tissues)**

0610007P22Rik, 0610009O03Rik, 0610012D09Rik, 0610039D01Rik, 1110012M11Rik, 1110017C15Rik, 1110020A09Rik, 1110020P15Rik, 1110035L05Rik, 1110049F12Rik, 1200003I07Rik, 1200003M09Rik, 1200011M11Rik, 1500011H22Rik, 1600010O03Rik, 1600013P15Rik, 1600020H07Rik, 1700025G04Rik, 1700030K09Rik, 1700065O13Rik, 1810032O08Rik, 1810034K20Rik, 1810037K07Rik, 1810055G02Rik, 2010107G23Rik, 2010305A19Rik, 2210019E14Rik, 2210409M21Rik, 2310002B06Rik, 2310003C23Rik, 2310007F12Rik, 2310022K01Rik, 2310022M17Rik, 2310033P09Rik, 2310035K24Rik, 2310042G06Rik, 2310047O13Rik, 2310061I09Rik, 2310065K24Rik, 2410001C21Rik, 2410002F23Rik, 2410131K14Rik, 2500002L14Rik, 2510048O06Rik, 2600011E07Rik, 2610024E20Rik, 2610301B20Rik, 2610318K02Rik, 2610507L03Rik, 2610528M18Rik, 2700060E02Rik, 2700082D03Rik, 2700085E05Rik, 2810405K02Rik, 2810417H13Rik, 2900001O04Rik, 2900002H16Rik, 2900010J23Rik, 3010001K23Rik, 3110002L15Rik, 3230401D17Rik, 3300001M20Rik, 4632423N09Rik, 4732479N06Rik, 4930422G04Rik, 4930429A22Rik, 4930429H24Rik, 4930570C03Rik, 4933407N01Rik, 5730405I09Rik, 5730420B22Rik, 5730494M16Rik, 5730502D15Rik, 5730593F17Rik, 5830434P21Rik, 5830457O10Rik, 6330527O06Rik, 6330577E15Rik, 6330578E17Rik, 6430706D22Rik, 6530403A03Rik, 6720401G13Rik, 6720463M24Rik, 9030227G01Rik, A130030D10Rik, A930031F18Rik, AA409316, AA415817, Aacs, Abcb8, Abce1, Acsl4, Actg2, Actr8, Aff3, Aggf1, Agpat1, Agpat5, AI854408, AI854635, Akap10, Alas1, Aldh3a2, Alg3, Als2cr2, Amotl2, Anapc2, Ankrd40, Anxa5, Ap2a1, Ap2b1, Apex1, App, Appbp2, Arbp, Arhgap11a, Arl10c, Arnt2, Arpc5l, Ars2, As3mt, Asna1, Asxl2, Atg4b, Atp5g2, Atp6v1g1, AU040320, Aurka, AW549877, AW555464, B130016L12Rik, B230380D07Rik, BB128963, BC003885, BC003993, BC012278, BC016423, BC026432, BC031441, BC033915, BC035295, BC050092, BC052040, BC055368, BC057627, BC059842, BC060267, BC060632, BC061237, Bcap31, Bcar3, Bcas2, Bccip, Bcor, Bcorl1, Bex4, Bicd2, Bnipl, Btrc, C130032J12Rik, C530028O21Rik, C78212, C80913, C86302, Cacybp, Calcoco1, Calm3, Caml, Casp6, Casp7, Cbx1, Cchcr1, Ccnb1, Cd200r2, Cd81, Cdc2l5, Cdc42se2, Cdca3, Cfl2, Chaf1b, Chmp2a, Chn2, Cluap1, Cmas, Cnbp1, Cnot3, Coro2b, Cox6c, Crabp2, Creb3, Crebbp, Crocc, Csnk1a1, Cstf2t, Ctbp2, Cugbp1, Cul3, Cxadr, Cyb5r4, Cyp4a28-ps, Cyp4f13, D10Ertd641e, D11Ertd707e, D11Wsu68e, D16Ertd480e, D2Ertd485e, D3Ucla1, D530033C11Rik, D5Ertd585e, D8Ertd812e, Dbf4, Dcamkl2, Dctn5Ddx46, Denr, Dhps, Diablo, Diap3, Dicer1, Dido1, Dlgap4, Dnajb10, Dnajc5, Dnajc6, Dnajc7, Dnttip2, Dpp8, Drap1, Dscr1, Dstn, Dus1l, E130309D02Rik, E2f4, Eaf2, Echs1, Edg1, Efemp2, Eif2s1, Eif3s1, Eif3s4, Eif4e, Eno1, Eps8l2, Erbb2, Esd, Etv6, Exosc7, F11r, , Fabp5, Farslb, Fbxw5, Fdps, Fh1, Fhl1, Flrt3, Fsd1, Fto, Fubp1, Fv1, G430022H21Rik, Gabrd, Galnt1, Gas8, Gcl, Gemin4, Ghitm, Git1, Gja7, Gmpr2, Gnl3, Golga7, Got1m, Gp38, Gpbp1, Gpc2, Gpc4, Gpc6, Gps2, Gpsm1, Grcc2f, , Gria4, Gsta4, Gstm4, Gstp1, Gtf3a, Gtf3c2, Gtpbp1, Gtpbp4, Guk1, H47, Hadhb, Hdgfrp2, Herpud1, Hisppd1, Hist1h2ae, Hlcs, Hlrc1, Hnrpl, Hps4, Hrbl, Hs6st1, Hspa4l, Hspa5, Huwe1, Hyal3, Hyou1, Iars, Ica1, Igf2bp3, Igsf11, Igsf4a, Il8rb, Ilvbl, Impa1, Jmjd2a, Khdrbs1, Kif23, Kirrel1, Klhl20, Kpna3L3mbtl2, Lap3, Laptm4a, Laptm4b, Lars2, Lig1, Lin9, Lincr, Lipg, Lmo4, LOC14433, LOC432823, Lor, Lrpap1, Lrrc20, Lrrc5, Madd, Maged1, Mageh1, Map3k4, Mapk12, Mapkapk5, Mesdc2, Metap2, Mfhas1, Mfng, MGC102419, Mgst3, Mid1, Midn, Mif, Minpp1, Mki67, Mkrn3, Mllt1, Mpa2l, Mphosph1, Mpp2, Mpst, Mrpl32, Mrpl37, Mrpl38, Mrpl44, Mrpl49, Mrpl9, Mrps11, Mtap1b, Mtbp, Mthfd1, Mum1, Mxd3, Mxd4, Mylk, Narg1, Nars, Nck1, Ndel1, Ndufa9, Ndufb11, Ndufs3, Nedd9, Nmt1, Noc4l, Nol1, Nol5a, Npc2, Nqo3a2, Nrbf1, Nsf, Nudcd3, Nufip1, Nup54, Nxf1, Ogfr, Osgep, Otub1, Oxsr1, Park7, Pbef1, Pbx1, Pbxip1, Pcca, Pcdhga1, Pcdhga10, Pctk1, Pcyox1, Pdhx, Pes1, Pgam1, Pgam2, Pgm2l1, Phf21a, Phf6, Phtf2, Phyh, Pigs, Pip5k1c, Pitpna, Pitpnb, Plekha6, Plekha7, Plekhg3, Polb, Pole, Polr2a, Polr2f, Polr2g, Polr2h, Pou6f1, Ppia, Ppp1ca, Ppp1cc, Ppp1r16a, Ppp2r4, Ppp5c, Pqbp1, Prdx2, Prdx4, Prei3, Prep, Prlpe, Prp19, Prpf3, Prpf38a, Prpf8, Psma5, Psmc2, Psmd14, Psme3, Ptpn9, Qars, Qdpr, Rab11b, Rabep2, Rad52, Ralgps2, Ran, Ranbp1, Rarsl, Rassf4, Rcc2, Rce1, Rcn1, Rec8L1, Recql4, Rgl2, Rhbdl7, Ric8, Rnaset2, Rnf8, Rpe, Rpl26, Rpl31, Rpl38, Rpl6, Rpo1-1, Rps14, Rps16, Rps17, Rps27l, Rps4x, Rrm1, Rsn, Scamp2, Scarb1, Scd3, Scnm1, Sdccag10, Sec13l1, Sec61a1, Sec61g, Sep15, Sephs2, Serpinh1, Sfrs10, Sfrs6, Sfrs8, Sh3kbp1, Shq1, Siah1b, Skp1a, Slc12a1, Slc15a2, Slc1a3, Slc25a11, Slc31a1, Slc35a4, Slc35b1, Slc39a3, Slc4a3, Smbp, Smg5, Smndc1, Smyd2, Snrpd3, Snx11, Snx17, Sod1, Sox11, Sp2, Spag5, Spag7, Spata5, Spcs2, Sphk2, Sppl3, , rebf2, Ss18l1, Stat3, Strap, Stxbp2, Suhw3, Suhw4, Supt4h1, Taf10, Taf9, Tanc1, Tars, Tbce, Tbrg1, Tceb3, Tde1, Tead2, Tes, Tfam, Tfdp2, Tfip11, Tgfbr1, Thrap1, Thrap6, Tinf2, Tlr11, Tlr13, Tlr5, Tm2d3, Tmem33, Tmem34, Tmem45b, Tnip1, Tnk2, Tnpo1, Tnrc9, Tomm20, Trappc3, Trappc4, Trim33, Trp53bp1, Trub2, Tsen2, Tsg101, Tspan32, Tspan6, Ttc4, Tuba1, Tuba2, Tubb2b, Tubgcp3, Txndc4, Txnip, Txnl1, Txnl4, Ube1c, Ube2r2, Ube2t, Uble1b, Ufm1, Urod, Usf1, Usf2, Usp15, Utp14b, Vangl2, Vapa, Vil2, Vps45, Wdr26, Wdr57, Wdr58, Wnt7a, Wrb, Wsb1, X83328, Xab2, Xbp1, Xpnpep1, Xpo7, Xylt2, Yme1l1, Zc3h3, Zfp111, Zfp259, Zfp263, Zfp330, Zfp346, Zfp397, Zfp459, Zfp61, Zfp661, Zfp74, Zfp94, Zfpl1, Zkscan1, Zmynd11
